# Supplementary figures and images for: An interpretable machine learning framework for dog breed inference and ancestry decomposition
Source: bioRxiv. 2026 Jun 4:2026.06.03.729926. Preprint. [Version 1] doi: 10.64898/2026.06.03.729926 (PMC13252084; doi:10.64898/2026.06.03.729926)

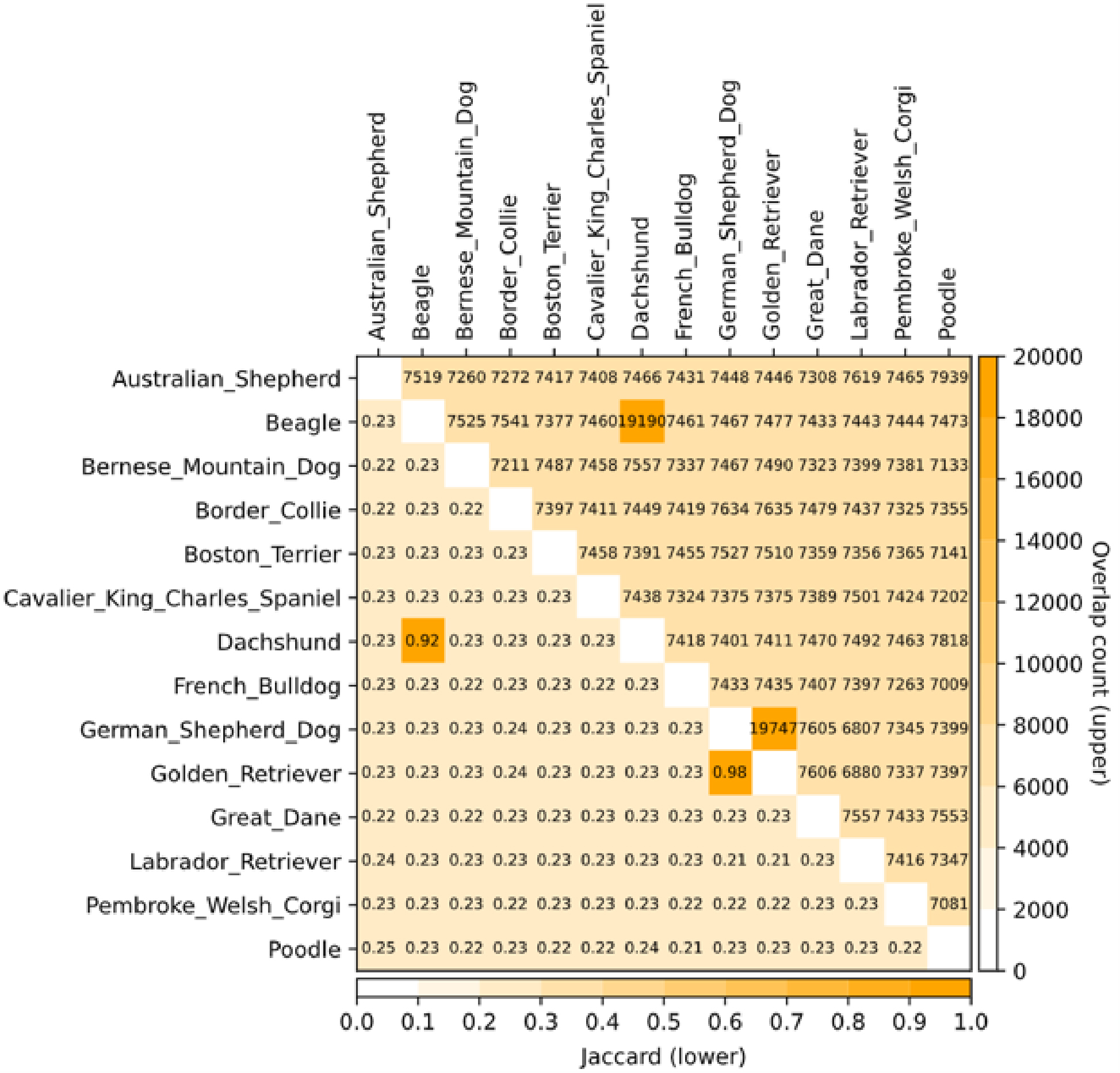

Supplement: Supplement 2 [file media-2.jpg]
